# Characteristics and comparison of colorectal cancer incidence in Beijing with other regions in the world

**DOI:** 10.18632/oncotarget.15598

**Published:** 2017-02-21

**Authors:** Zhongmin Li, Lei Yang, Changzheng Du, Xuedong Fang, Ning Wang, Jin Gu

**Affiliations:** ^1^ Department of Gastrointestinal Colorectal and Anal Surgery, China-Japan Union Hospital of Jilin University, Changchun, China; ^2^ Key Laboratory of Carcinogenesis and Translational Research (Ministry of Education/Beijing), Beijing Office for Cancer Prevention and Control, Peking University Cancer Hospital and Institute, Beijing, China; ^3^ Key Laboratory of Carcinogenesis and Translational Research (Ministry of Education/Beijing), Department of Gastrointestinal Surgery Peking University Cancer Hospital and Institute, Beijing, China; ^4^ Department of General Surgery, Peking University Shougang Hospital, Beijing, China; ^5^ Tsinghua-Peking Joint Center for Life Sciences, Peking University, Beijing, China

**Keywords:** Beijing, population-based, colorectal cancer, incidence, trend

## Abstract

**Background:**

Population-based epidemiologic studies about colorectal cancer are lacking in China. This study aims to provide a basis for colorectal cancer screening and prevention, through analysis and comparisons the characteristics of the trends in colorectal cancer incidence in Beijing and selected representative regions.

**RESULTS:**

The annual incidence rate in Beijing region increased significantly, from 9.40/100,000 in 1998 to 18.61/100,000 in 2012. The stratified rate showed that the incidence of distal colon adenocarcinoma increased substantially in men, especially in those aged > 75 years and residing in urban areas. Although the incidence rate in Beijing is still lower than in Shanghai, Jiashan, and Hong Kong in China, it is increasing rapidly. Further, the incidence rate in Beijing is lower than in New York, Oxford and Osaka, but higher than in Mumbai and Kyadondo. The incidence trend in Beijing is increasing especially in older groups, while in other regions such as New York, it is decreasing in these age groups.

**Materials and Methods:**

Colorectal cancer incidence data were obtained from Beijing Cancer Registry and Cancer Incidence in Five Continents Plus database. All incidence rates were age-standardized according to Segi's world population. Incidence trends were characterized by calculating the annual percent changes using the Joinpoint Regression Program.

**Conclusions:**

Compared with other regions, Beijing has a medium level of colorectal cancer incidence, however, it is increasing significantly. There are obvious differences in the cancer subsite, sex and age distributions between Beijing and other regions. Prevention and screening of colorectal cancer in Beijing should be strengthened.

## INTRODUCTION

The incidence of malignant tumors in China has increased significantly in recent years, from 184.81/100,000 in 1989 to 286.69/100,000 in 2008 [1]. An estimated 4,292,000 new cases and 2,814,000 cancer-associated deaths were predicted to occur in China in 2015 [2]. The latest data shows that there were an estimated 310,244 new cases diagnosed with colorectal cancer in 2011, accounting for 9.20% of all newly diagnosed cancer cases [3]. Accordingly, colorectal cancer is becoming a major threat to the lives and health of Chinese people.

Population-based epidemiologic studies about colorectal cancer are lacking in China as compared with in developed countries. The Beijing Cancer Registry, which is part of the International Association of Cancer Registries (IACR), has collected population-based cancer incidence data since 1976 and these data are included in the Cancer Incidence in Five Continents (CI5) database, which is published by the International Agency for Research on Cancer (IARC). Since 1998, the registry surveillance coverage has expanded from 8 to 16 districts and residents covering changed from 7 to 12 million. Medical records of the newly diagnosed cancer inpatients were monthly required to report to the Beijing Cancer Registry from all the 138 medical hospitals in Beijing. The population, economic level, and city size of Beijing are very representative of China. Using population-based cancer registry data in Beijing between 1998 and 2012, we investigated the incidence trend of colorectal cancer and analyzed its characteristics by sex, subsite, age, and other aspects. We further compared the incidence data with the data from other regions of China and the world. The aim of this study was to analyze the differences in incidence between Beijing and other regions in order to provide more information for colorectal cancer screening and prevention.

## RESULTS

### Characteristics of colorectal cancer incidence in Beijing

**Figure 1:**
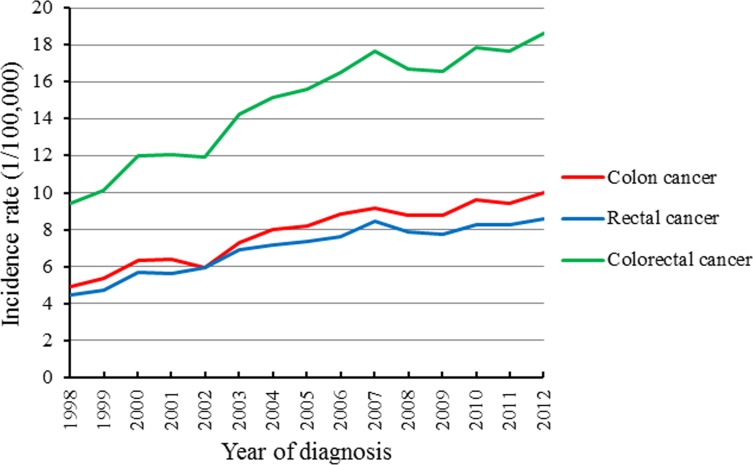
Trends of colorectal cancer incidence rate in Beijing between 1998 and 2012

**Figure 2:**
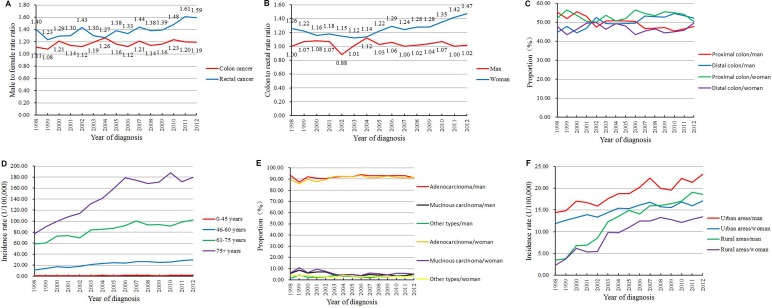
Trends of colorectal cancer incidence in Beijing by (**A**) male to female rate ratio; (**B**) colon to rectal rate ratio; (**C**) proximal and distal colon; (**D**) age; (**E**) pathological type; (**F**) areas (urban/rural).

Between 1998 and 2012, 43,990 cases of colorectal cancer were registered in the Beijing Cancer Registry (Table 1). As shown in Figure 1, the annual incidence rate of colorectal cancer in Beijing increased significantly, from 9.40/100,000 in 1998 to 18.61/100,000 in 2012. The rectal cancer incidence increased faster than the colon cancer incidence. The most significant increase was noted from 1998 to 2005, during which the annual percent change (APC) was 7.7%. After 2005, the incidence rate increased slowly each year, with APC of approximately 1.8% (Table 2). The incidence rate in men was much higher than that in women, this was especially exhibited in cases of rectal cancer, for which the male to female ratio was increased to 1.61 in 2011, whereas the male to female ratio of colon cancer remained relatively stable (Figure 2A). Although the incidence rate of colon cancer was slightly higher than that of rectal cancer (Figure 1), the colon to rectal ratio in women increased substantially in recent years, while the ratio in men did not change obviously (Figure 2B). In men, proximal colon cancer was more common than distal colon cancer before 2001, but the trend reversed after 2006. The proportion of distal colon cancer was increasing annually, rising to 54.69% in 2010. The proportion in women did not obviously change (Figure 2C). Next, all cases studied were classified into four groups according to the age at diagnosis. The incidence rates were increasing in all groups over time, especially in the 46∼60 years group before 2004 and in the 75+ years group before 2006 (Figure 2D). After 2006, the incidence rates in individuals older than 60 years remained stable, while those in the groups aged younger than 60 years continued to increase slowly (Table 3).

**Table 1:** Clinicopathological characteristics of patients diagnosed as colorectal cancer in Beijing between 1998 and 2012

Characteristic	Variable	Case
Gender	Male	24165
	Female	19825
Site	Colon	23563
	Rectal	20427
Colon	Proximal	7588
	Distal	7305
	unknown	8670
Pathologic classification	Adenocarcinoma	30845
	Mucinous carcinoma	1716
	Other types	1014
	unknown	10415

**Table 2:** The annual percent change of colorectal cancer incidence rate in Beijing between 1998 and 2012

Cancer site	Trend			
	Year	APC (%)	95% CI	*P*
Colon cancer	1998–2006	7.1	[5.1, 9.1]	0.00
	2006–2012	1.7	[−1.3, 4.7]	0.23
Rectal cancer	1998–2004	8.6	[6.2, 11.1]	0.00
	2004–2012	1.9	[0.4, 3.4]	0.02
Colorectal cancer	1998–2005	7.7	[5.7, 9.7]	0.00
	2005–2012	1.8	[−0.1, 3.8]	0.04

**Table 3:** The annual percent change of colorectal cancer incidence rate by age in Beijing between 1998 and 2012

Age	Trend			
	Year	APC (%)	95% CI	*P*
0–45 years	1998–2012	2.8	[1.3, 4.4]	0.00
46–60 years	1998–2004	11.5	[7.6, 15.5]	0.00
	2004–2012	2.6	[0.3, 5.0]	0.03
61–75 years	1998–2006	5.8	[3.8, 7.8]	0.00
	2006–2012	0.7	[−2.2, 3.7]	0.61
75+ years	1998–2006	10.3	[9.0, 11.5]	0.00
	2006–2012	0.3	[−1.5, 2.1]	0.71

Adenocarcinoma was the most common pathological type, accounting for about 90% of all cases. As shown in Figure 2E, the incidence of adenocarcinoma is slowly increasing, whereas that of mucinous carcinoma is decreasing. The incidence of colorectal cancer was much higher in urban areas. However, the incidence rate in rural areas was found to increase more rapidly, indicating that the gap between urban and rural areas is gradually being reduced (Figure 2F). The incidence in rural areas increased dramatically from 1998 to 2004, with an APC of 26.2% in men and 25.4% in women. After this period, the APC in rural areas was reduced to 2.7% in women and 3.5% in men.

### Comparison with representative regions in China

**Figure 3:**
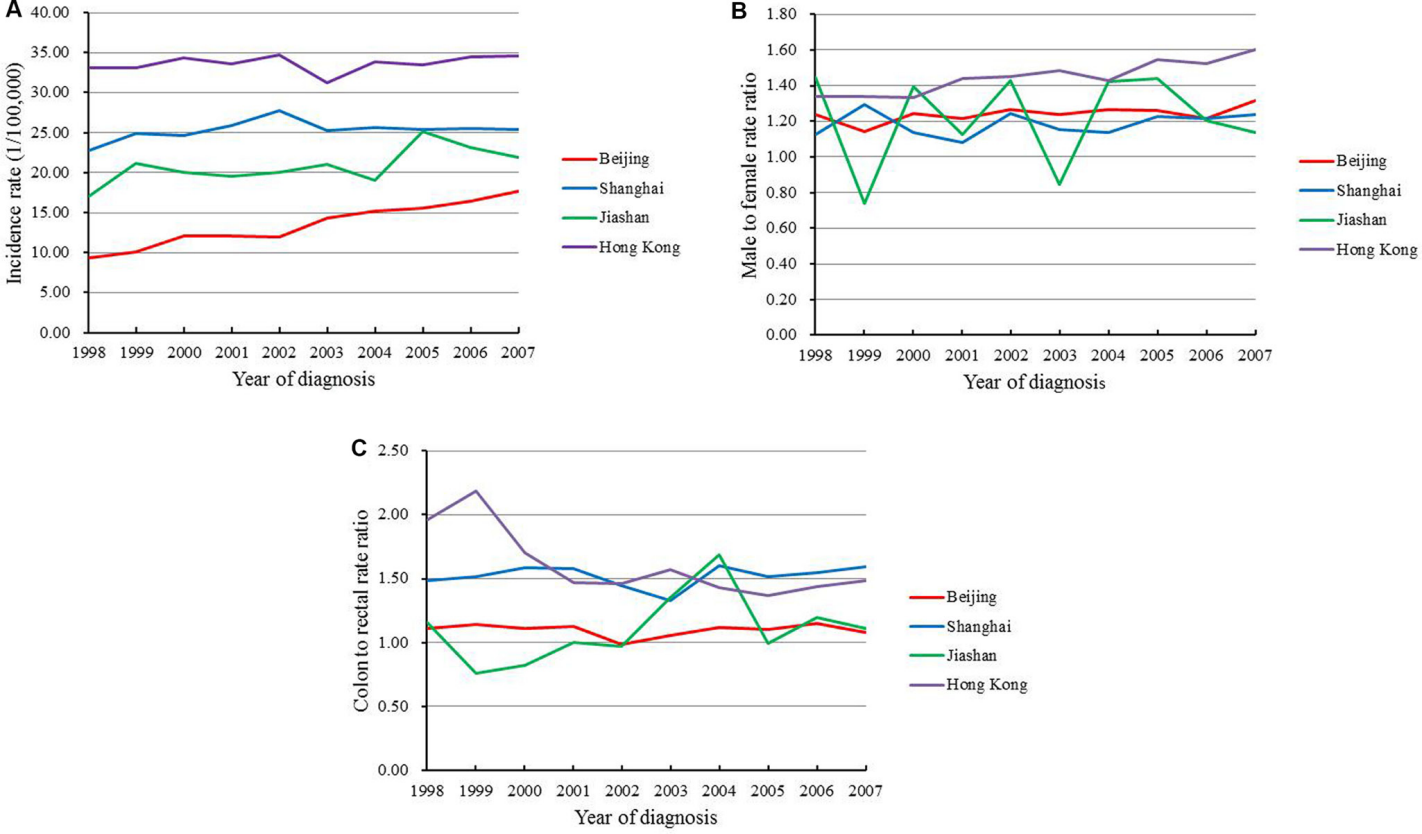
Trends of (**A**) colorectal cancer incidence rate; (**B**) male to female rate ratio; (**C**) colon to rectal rate ratio in Chinese four representative regions between 1998 and 2007.

The colorectal cancer incidences of Hong Kong, Shanghai, and Jiashan were compared with the data from Beijing between 1998 and 2007. During the study period, Beijing and Jiashan showed increase of incidences, with APC of 7.1% and 2.4%, respectively, while the rates remained stable in Hong Kong and Shanghai (Figure 3A). High male to female rate ratios were found in Beijing, Shanghai and Hong Kong (Figure 3B). Similar to Shanghai and Hong Kong, a high colon to rectal rate ratio was seen in Beijing, although it was still the lowest of the examined regions. The ratio decreased in Hong Kong before 2001, but subsequently remained stable, similar to in Beijing and Shanghai. The incidence of colon cancer also exceeded that of rectal cancer in Jiashan after 2002 (Figure 3C).

In the 0–45 years group, the annual incidence gradually increased in Beijing, while that in Shanghai decreased by 3.4% each year. In Jiashan, the incidence decreased before 2002, followed by obvious increase of 16.2% each year thereafter. No significant change was seen in Hong Kong. In the 46–60 years group, all regions except Jiashan experienced yearly increase. In the 61–75 years group, the incidences increased in Beijing and Jiashan, while they remained stable in Hong Kong and Shanghai. In the 75+ years group, the incidence increased in all regions. The incidence remained the highest, although stable, in Hong Kong, while Beijing experienced a fast increase with an APC of 9.8% (Table 4).

**Table 4:** The annual percent change of colorectal cancer incidence rate by age in Chinese four representative regions

Age	Area	Trend			
		Year	APC (%)	95%CI	*P*
0–45 years	Beijing	1998–2007	3.8	[0.6, 7.0]	0.02
	Shanghai	1998–2007	−3.4	[−5.6, −1.1]	0.01
	Jiashan	1998–2002	−5.1	[−12.8, 3.3]	0.17
		2002–2007	16.2	[9.5, 23.4]	0.00
	Hong Kong	1998–2007	−0.1	[−2.5, 2.4]	0.91
46–60 years	Beijing	1998–2007	9.2	[6.9, 11.5]	0.00
	Shanghai	1998–2007	2.2	[0.4, 4.0]	0.02
	Jiashan	1998–2007	2.5	[−2.5, 7.9]	0.29
	Hong Kong	1998–2007	2.5	[1.6, 3.4]	0.00
61–75 years	Beijing	1998–2007	5.8	[4.4, 7.2]	0.00
	Shanghai	1998–2007	0.3	[−0.7, 1.3]	0.52
	Jiashan	1998–2007	2.3	[−1.5, 6.3]	0.20
	Hong Kong	1998–2007	0.3	[−1.0, 1.6]	0.60
75+ years	Beijing	1998–2007	9.8	[8.7, 10.9]	0.00
	Shanghai	1998–2007	3.4	[1.0, 5.9]	0.01
	Jiashan	1998–2007	3.8	[−2.5, 10.5]	0.21
	Hong Kong	1998–2007	−0.1	[−1.0, 0.8]	0.80

### Comparison with representative regions worldwide

**Figure 4:**
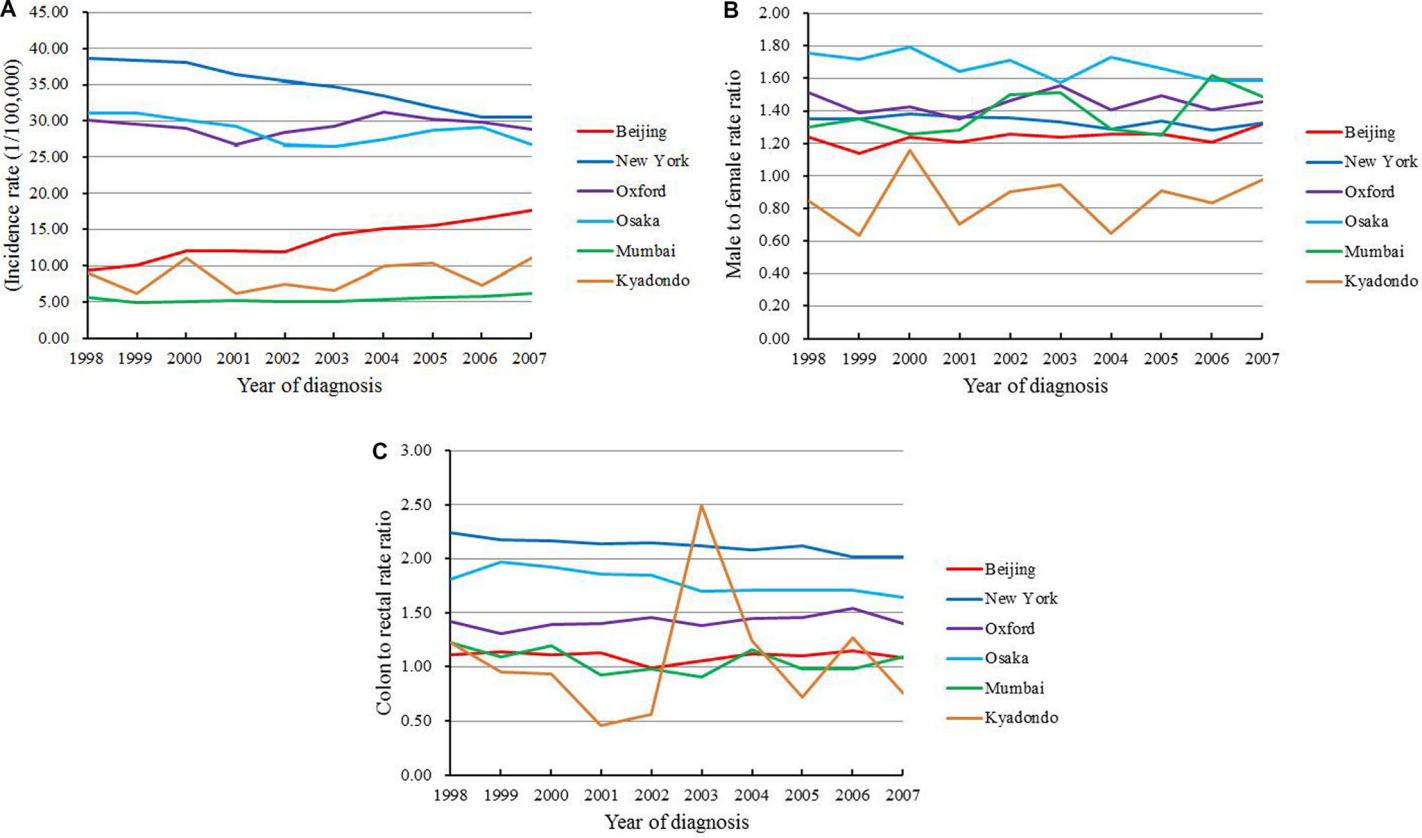
Trends of (**A**) colorectal cancer incidence rate; (**B**) male to female rate ratio; (**C**) colon to rectal rate ratio in international representative regions between 1998 and 2007.

The trends of colorectal cancer incidence in international representative regions between 1998 and 2007 were compared with the data of Beijing. By 2007, Beijing ranked in the middle, with lower incidence than New York (United States), Oxford (United Kingdom), and Osaka (Japan), but higher than Kyadondo (Uganda) and Mumbai (India). The incidence rate increased fast by 7.1% every year in Beijing, while it decreased by 2.9% yearly in New York. Oxford experienced a decreased of 3.9% each year before 2001, whereas the incidence increased by 4.6% each year from 2001 to 2004, and then decreased again by 2.3% each year after 2004. On the other hand, in Osaka, the incidence decreased slightly during these years, with an APC of 1.3%. The incidence rate of Mumbai remained low and stable before 2003, and then it increased obviously by 5.2% each year. No significant change was observed in Kyadondo (Figure 4A).

The incidence rate in men was higher than women in all of these regions, except in Kyadondo, where colorectal cancer was more common in women. The male to female rate ratio was highest in Osaka, while Beijing ranked the 2nd lowest. This ratio decreased obviously during the study period in Osaka, while it gradually increased in Mumbai (Figure 4B). The colon to rectal rate ratio was the highest in New York, with the incidence of colon cancer being twice as high as that of rectal cancer, followed by Osaka and Oxford. Beijing and Mumbai exhibited the lowest ratios. New York and Osaka experienced decreases in the ratios from 1998 to 2007, whereas the ratio was increasing in Oxford. The ratios remained stable in Beijing and Mumbai, while more variable ratios were seen in Kyadondo during the study period (Figure 4C).

In the 0-45 years group, the highest incidence of colorectal cancer was seen in New York, while the lowest in Mumbai and Kyadondo. The incidence rates in this age group increased in New York, Oxford and Beijing over time. In the 46–60 years group, New York and Osaka showed the highest incidences, while Mumbai showed the lowest. The rates of Beijing and Kyadondo were similar and gradually increased each year. In the 61–75 years group, the incidence rate in New York decreased by 4.8% each year, followed by Oxford and Osaka. On the contrary, the incidence rate was on the rise in Beijing. In Mumbai, the incidence rate decreased slightly before 2000, then increased obviously by 4.0% each year, whereas it remained stable in Kyadondo. In the 75+ years group, the incidence rate in New York decreased by 3.7% each year, and a similar decrease was seen in Osaka. In Oxford, the rate increased slightly before 2004, then gradually decreased. On the contrary, it increased obviously in Beijing and Mumbai (Table 5).

**Table 5:** The annual percent change of colorectal cancer incidence rate by age in international representative regions

Age	Area	Trend			
		Year	APC (%)	95% CI	*P*
0–45 years	Beijing	1998–2007	3.8	[0.6, 7.0]	0.02
	New York	1998–2007	2.7	[1.4, 4.1]	0.00
	Oxford	1998–2007	3.5	[−0.7, 7.9]	0.09
	Osaka	1998–2007	−1.0	[−4.0, 2.0]	0.45
	Mumbai	1998–2002	4.2	[−3.7, 12.7]	0.01
		2002–2005	−6.4	[−27.0, 20.0]	0.34
		2005–2007	18.0	[−8.0, 51.3]	0.02
	Kyadondo	1998–2007	0.1	[−10.4, 11.9]	0.98
46–60 years	Beijing	1998–2007	9.2	[6.9, 11.5]	0.00
	New York	1998–2007	−0.1	[−0.8, 0.6]	0.68
	Oxford	1998–2007	0.7	[−0.7, 2.0]	0.29
	Osaka	1998–2007	−0.1	[−2.1, 1.9]	0.87
	Mumbai	1998–2007	−0.4	[−3.2, 2.5]	0.75
	Kyadondo	1998–2007	6.6	[2.4, 10.9]	0.01
61–75 years	Beijing	1998–2007	5.8	[4.4, 7.2]	0.00
	New York	1998–2007	−4.8	[−5.5, −4.0]	0.00
	Oxford	1998–2001	−6.0	[−11.2, −0.5]	0.04
		2001–2004	5.1	[−6.2, 17.8]	0.12
		2004–2007	−3.5	[−8.8, 2.2]	0.03
	Osaka	1998–2002	−3.9	[−6.2, −1.5]	0.02
		2002–2005	2.0	[−5.5, 10.1]	0.39
		2005–2007	−3.0	[−10.2, 4.7]	0.23
	Mumbai	1998–2000	−5.8	[−19.6, 10.4]	0.38
		2000–2007	4.0	[1.8, 6.2]	0.01
	Kyadondo	1998–2007	1.2	[−7.9, 11.3]	0.77
75+ years	Beijing	1998–2007	9.8	[8.7, 10.9]	0.00
	New York	1998–2007	−3.7	[−4.3, −3.1]	0.00
	Oxford	1998–2001	−1.2	[−10.2, 8.7]	0.65
		2001–2004	4.2	[−14.0, 26.2]	0.45
		2004–2007	−3.0	[−11.9, 6.7]	0.30
	Osaka	1998–2007	−1.1	[−2.6, 0.5]	0.16
	Mumbai	1998–2007	3.7	[1.7, 5.7]	0.00
	Kyadondo	1998–2007	2.3	[−13.5, 21.1]	0.76

## DISCUSSION

The characteristics of the annual colorectal cancer incidence in Beijing between 1998 and 2012 were investigated in this study. The incidence rate increased rapidly in all age groups, with the most significant increase seen in rectal cancer in men, although colon cancer remained the major type.

In Beijing, a shift in the location of cancer from the proximal to distal colon was identified in men. This trend differed from the findings reported from North America and other high-risk areas, where a shift from the distal to proximal colon was conversely observed. In 1966, Axtell et al. first reported a shift from left-sided to right-sided colorectal cancer in the United States [4], a finding that was subsequently confirmed by several epidemiological studies [5, 6]. The proportion of proximal colon cancer increased by 6% from 1973 to 1997 in the United States [6]. Similar subsite distributions were also noted in other countries. In New Zealand, Shah et al. identified a left-sided to right-sided shift of colon cancer in women over the age of 65 [7]. The reason for this change in subsite distribution is not entirely clear, but may be partly due to the wide use of colonoscopy and sigmoidoscopy to detect precancer polyps. The survival of proximal and distal colon cancer differ [8], and the shift of colon cancer location may help in studying these differences in mortality. Routine colonoscopy screening was carried out relatively late in Beijing, and only in limited areas. This may explain why a left-to-right shift was not observed in this study.

Significant increases in incidence were observed in both urban and rural areas in Beijing. The gap was obvious before 2004, but slowly narrowed thereafter. The difference between urban and rural areas might be associated with different economic levels, living habits, and sanitary conditions [9], and urbanization will help narrow the gap between urban and rural areas. On the contrary, the incidence rate is higher in low-income areas in developed countries, such as America, where low socioeconomic status has been reported as a risk factor for colorectal cancer, the incidence of the most deprived areas is approximately 20% higher than that of the least deprived areas [10]. In developed countries, obesity, unhealthy diet habits, physical inactivity and cigarette smoking are more common in low socioeconomic status areas [11]. However, in developing countries such as China, obesity, cigarette smoking, high-fat and high-calorie diets, and red meat or processed meat intake are more common in urban areas. This might be the reason why the colorectal cancer incidence of urban areas was higher in China, which was different from the findings in developed countries.

In the four representative regions of China, Hong Kong had the highest incidence of colorectal cancer, followed by Shanghai and Jiashan, while Beijing had the lowest incidence. However, the incidence is increasing rapidly in Beijing. With the rapid economic development after the second world war, the total energy and western-type food intake increased in Hong Kong, and this might have contributed to the rapid increase in colorectal cancer incidence in the last century [12], as a result, the incidence in Hong Kong was the highest among the four regions. But the economy of Hong Kong grew slowly between 1998 and 2007. The Gross Domestic Product (GDP) grew from 168.886 billion dollar to 211.597 billion dollar during this period, and it even declined in a few years [13]. This might explain why the incidence remained stable in this study. Being the largest economic and industrial city of China, Shanghai has a fairly high incidence of colorectal cancer, which increased obviously between 1972 and 1994 [14]. In this study, the incidence was found to reach up to 25.38/100,000 in 2007. This is likely mainly associated with the changes in dietary habits [15]. Due to the high incidence of cancer, some preventive measures have been taken in Shanghai, such as cancer screening, diet education, tobacco control. The incidence rate of lung cancer was decreasing attributed to decreased tobacco use in urban Shanghai [16]. A local colorectal cancer screening program using biennial fecal occult blood testing (FOBT), followed by colonoscopy in positive patients, has been carried out in Shanghai since 2012, and this program will influence the incidence trend in future. Jiashan is widely known to have high incidence of colorectal cancer and is ranked first at the county level in China. Many case control studies about the etiology of colorectal cancer have been carried out in Jiashan since 1980, with a history of intestinal polyps, drinking polluted water, and organic chlorine pollution identified as major risk factors [17, 18]. The incidence rate of Jiashan kept increasing between 1998 and 2007, which might associate with the intensive screening that carried out since late 1980s. More colorectal cancer was detected at an early time, so the incidence increased during this short period.

Internationally, the incidence rates in New York, Oxford and Osaka were higher than those in Beijing, Mumbai and Kyadondo, indicating obvious differences between different areas. However, the incidence gap was gradually narrowing. The incidence has decreased in New York, Oxford and Osaka in recent years. Meanwhile, the incidence has increased rapidly in Beijing, while the incidence rates in Mumbai and Kyadondo have also began to increase slightly, although they are still low. Routine colorectal cancer screening has been reported as the main reason for the lower incidence of colorectal cancer in the United States [19]. From 1987 to 2010, the percentage of adults aged over 50 years undergoing colorectal cancer screening was increased from from 34.8% to 66.1%. By colorectal screening, a reduction of approximately 550,000 cases of colorectal cancer was observed during this period [20], mainly as a result of the intestinal polyps that might progress to colorectal cancer being removed by colonoscopy [21, 22]. Developing countries such as China and India lack colorectal cancer screening guidelines. Although the colorectal cancer incidence in Beijing was at a moderate level compared to in other countries worldwide, the rate has increased significantly and has overtaken the rates of Mumbai and Kyadondo. Thus, the need for prevention and screening of colorectal cancer in Beijng has become very critical. In the United States and Europe, the incidence of colon cancer is obviously higher than rectal cancer. However, rectal cancer accounts for more than 50% of cases in Asia and South America, where the colorectal cancer incidence is relatively low [23]. In China, rectal cancer accounted for more than 70% of colorectal cancers in the 1970s, although this proportion has decreased in recent years [24]. In this study, the incidence rates of colon cancer were found to be higher than those of rectal cancer in all four regions, indicating that colon cancer has become more common in China. Changes in life style and the screening procedures might have contributed to the increase in the proportion of colon cancer [14, 23]. In the present study, the incidence trends of the different age groups varied. The incidence rates of all age groups were increasing in Beijing, especially in the 75+ age group. In New York, the incidence was found to be increasing in people aged less than 45 years old, and was decreasing obviously in people older than 60 years. The reason for the incidence of colorectal cancer is increasing in young people in the United States is unclear, but it might be related to the increasing rates of obesity and diabetes in the younger population. On the other hand, the decrease in incidence in old people has been reported to be associated with improved colorectal cancer screening [25].

Many factors have been reported to be associated with colorectal cancer. One study from Shanghai showed that the increased incidence of colorectal cancer in Shanghai was closely related to the increased intake of meat [26]. From 1993 to 2009, the prevalence of overweight and obesity in Chinese adults significantly increased. The rate of obesity increased from 2.9% to 11.4% in men, and from 5.0% to 10.1% in women [27]. Hence, obesity may be an important factor related to the increase in colorectal cancer incidence in China. In 2010, there were an estimated 301 million current smokers in China, which made China the largest consumer of tobacco in the world [28]. One study in the Chinese population showed that smoking could significantly increase the risk of colorectal cancer specific mortality by about 10% as compared with non-smokers [29]. A previous study from Jiangsu Province, China, showed a significant correlation between alcohol consumption and colorectal cancer [30]. Furthermore, environmental pollution and accelerated aging of the population are also serious problems that China is currently facing. Studies have shown that approximately 350,000 to 500,000 people die prematurely every year as a result of outdoor air pollution in China [31]. At present, about 12% of the Chinese population are aged more than 60 years old, and this aged proportion will increase to 24% by 2035 [32]. Our study showed that the incidence of colorectal cancer increased obviously by age. Therefore, we expect that an overall increase in the incidence of colorectal cancer will occur in China with the increasing elderly population.

We speculate that the rapid increase of colorectal cancer incidence in Beijing can be attributed to the following reasons: 1) the accelerated industrialization; 2) rapidly increasing economic level; 3) gradually popular western diet habits; 4) increasing rates of obesity, smoking and drinking; and 5) increasingly aging population. Further, the aggravating environmental pollution could be another reason. We plan to carry out a serious of case-control studies in the next step to confirm the risk factors of colorectal cancer. In order to reduce the incidence of colorectal cancer, we need to avoid the risk factors and take some effective control measures. Colorectal cancer screening can effectively reduce the incidence and mortality of colorectal cancer. We suggest that the government should increase investment in health care and colorectal cancer screening should be included in medical insurance as the United States. The incidence of colorectal cancer was extremely low in the people below 45 years old in this study. We recommend that people start colorectal cancer screening from 45 years old.

There are some limitations in this study. First, because most of the cases in this study did not have definite staging, the incidence trend by stage was not included in the analysis, even though staging is believed to be an important epidemiological characteristic [33]. Second, the Beijing Cancer Registry is a different data sources from CI5plus, and the incidence data of the other regions were available only from 1998 to 2007, which might have impacted on the results. Finally, the specific reason for the rapid increase of colorectal cancer incidence in Beijing need to be further explored in addition to certain risk factors such as the changes in economic level, diet, and living habits.

In conclusion, the incidence of colorectal cancer in Beijing increased obviously between 1998 and 2012. During this period, the location of colon cancer in men showed a shift from the proximal to distal colon. The incidence rate in urban areas was much higher than rural areas. The incidence of colorectal cancer in Beijing has increased significantly, although it is still lower than Hong Kong, Shanghai, and Jiashan. Compared with other cities in the world, Beijing has a medium level of colorectal cancer incidence. Thus, the prevention, screening, and early diagnosis of colorectal cancer should be strengthened in Beijing, especially for people older than 60 years and living in urban areas.

## MATERIALS AND METHODS

We selected a number of representative regions both in China and worldwide as a means to compare the epidemiological characteristics of colorectal cancer with those of Beijing. Colorectal cancer incidence data of Beijing were derived from the Beijing Cancer Registry between 1998 and 2012. The incidence data of other regions were obtained from IARC's Cancer Incidence in Five Continents Plus (CI5 plus) database between 1998 and 2007 [34]. Colorectal cancer incidence data were coded according to the 10th edition of the International Classification of Diseases. The proximal colon included the cecum, ascending colon, hepatic flexure, transverse colon, and splenic flexure; the distal colon included the descending colon and sigmoid colon; and the rectum included the rectosigmoid junction, rectum, and anus, which refer to tumors within 15cm from the anal verge. Colorectal cancer was classified as adenocarcinoma, mucinous carcinoma, and other types, including carcinoid tumor and adenosquamous carcinoma, among others, according to the International Classification of Diseases for Oncology, 3rd edition.

The incidence data of Beijing were investigated based on the quality criterion of CI5 by the IARC [35]. The crude rates (CR) and world age-standardized rates (WASR) were calculated stratified by sex, subsite, age, pathologic classification, and areas (urban/rural). Temporal trends were characterized by calculating the annual percent change (APC) of WASR using Joinpoint 4.1.1.1 Regression Program. APC is used to measure the trend or change in rate over a single year. It is the average annual rate of change within the time series selected. All incidence rates were age-standardized to Segi's world population and expressed per 100,000 person-years. The male to female rate ratio was defined as the ratio of the incidence rates in men vs. women. The colon to rectal rate ratio was defined as the ratio of the incidence rates of colon cancer vs. rectal cancer.

## Abbreviations

IACR: International Association of Cancer Registries
CI5: Cancer Incidence in Five Continents
IARC: International Agency for Research on Cancer
CR: Crude rates
WASR: World age-standardized rates
APC: Annual percent change
GDP: Gross Domestic Product
FOBT: Fecal occult blood testing.
